# Epithelial-Mesenchymal Transition (EMT) and Regulation of EMT Factors by Steroid Nuclear Receptors in Breast Cancer: A Review and *in Silico* Investigation

**DOI:** 10.3390/jcm5010011

**Published:** 2016-01-19

**Authors:** Ioannis A. Voutsadakis

**Affiliations:** 1 Division of Medical Oncology, Department of Internal Medicine, Sault Area Hospital, Sault Ste Marie, ON P6B 0A8, Canada; ivoutsadakis@yahoo.com or ivoutsadakis@nosm.ca; Tel.: +1-705-759-3434; 2 Division of Clinical Sciences, Northern Ontario School of Medicine, Sudbury, QC P3E 2C6, Canada

**Keywords:** Steroid Nuclear Receptors, Epithelial Mesenchymal Transition, Mesenchymal Epithelial Transition, ERα, PR, AR, ERβ, GR, MR, breast cancer

## Abstract

Steroid Nuclear Receptors (SNRs) are transcription factors of the nuclear receptor super-family. Estrogen Receptor (ERα) is the best-studied and has a seminal role in the clinic both as a prognostic marker but also as a predictor of response to anti-estrogenic therapies. Progesterone Receptor (PR) is also used in the clinic but with a more debatable prognostic role and the role of the four other SNRs, ERβ, Androgen Receptor (AR), Glucocorticoid Receptor (GR) and Mineralocorticoid Receptor (MR), is starting only to be appreciated. ERα, but also to a certain degree the other SNRs, have been reported to be involved in virtually every cancer-enabling process, both promoting and impeding carcinogenesis. Epithelial-Mesenchymal Transition (EMT) and the reverse Mesenchymal Epithelial Transition (MET) are such carcinogenesis-enabling processes with important roles in invasion and metastasis initiation but also establishment of tumor in the metastatic site. EMT is governed by several signal transduction pathways culminating in core transcription factors of the process, such as Snail, Slug, ZEB1 and ZEB2, and Twist, among others. This paper will discuss direct regulation of these core transcription factors by SNRs in breast cancer. Interrogation of publicly available databases for binding sites of SNRs on promoters of core EMT factors will also be included in an attempt to fill gaps where other experimental data are not available.

## 1. Introduction

Breast cancer is the most common cancer in women and a majority of cases (about three-fourths) express the Estrogen Receptor (ERα). Most of these cancers co-express different degrees of the Progesterone Receptor (PR) and the Androgen Receptor (AR). Some of the ER-negative breast cancers express the AR and constitute a special category of those ER-negative cancers which have commonly apocrine features on pathologic examination [1]. All three receptors, together with Glucocorticoid Receptor (GR), Mineralocorticoid Receptor (MR) and a second nuclear receptor for estrogens termed ERβ, comprise the steroid receptor sub-family of nuclear transcription factors. They display a common protein structure with conserved domains, not only within the steroid receptor sub-family but also with non-steroid receptors [2]. These domains include an amino-terminal activation domain (Activation Function 1, AF-1), a DNA-binding domain, a hinge region and a carboxy-terminal ligand-binding domain [3]. Response sequences on target gene promoters have similarities amongst the Steroid Nuclear Receptors (SNRs) [4]. The various SNRs regulate different sets of genes due to additional factors such as the availability of their ligands, their own expression and post-translational modifications that regulate their transcriptional activity and the availability of co-regulators such as co-activators, co-repressors and pioneer factors that shape the overall effect of each SNR on the whole genome scale in a particular cell by modifying the chromatin landscape and the ability of SNRs to interact with it [5,6,7].

The roles of Epithelial to Mesenchymal Transition (EMT) and the reverse Mesenchymal to Epithelial Transition (MET) processes as central enabling capabilities in cancer invasion and metastasis have been confirmed over the last several years and involve the activity of a core set of transcription factors (TFs) activated by signal transduction pathways in neoplastic cells [8,9]. The following sections will discuss available evidence of direct regulation of transcription of EMT core transcription factor genes by SNRs in breast cancer. Indirect regulations are also important but will not be considered in detail in this overview, except in situations where their discussion will elucidate the discussion of direct regulations. A further inquiry through publicly available promoter databases will be reported on direct regulations where other experimental data are lacking in order to guide future investigations.

## 2. Epithelial to Mesenchymal Transition (EMT) and Mesenchymal to Epithelial Transition (MET) in Breast Cancer

EMT is a process that physiologically takes place during normal embryonic development and in adult tissue injury repair. In contrast to these two physiologic conditions where EMT serves normal functions, cancer is a pathologic condition where EMT occurs [8]. During EMT in cancer, transformed epithelial cells lose epithelial membrane adhesions, invade through the epithelium basement membrane and acquire the ability to move on freely in neighboring tissue or to metastasize to distant organs using vascular routes. Cells undergoing EMT, in addition to losing inter-cellular junctions, lose epithelial cell polarity, gain a fibroblast-like shape, down-regulate epithelial markers such as E-cadherin, claudins, occludins and cytokeratins, and up-regulate mesenchymal markers such as S100A4 (also called FSP1(Fibroblast-Specific Protein 1)), vimentin and cadherin N [10].

EMT occurring in cancer may be incomplete both in the individual cell and the cell population level and only part of the EMT markers may be expressed in small sub-sets of cancer cells [11]. Incomplete EMT, as, for example, seen during the process of collective migration, allows cells to detach from the epithelial site, acquire some mesenchymal features, but still move as small groups of few cells without losing adhesions between the members of the group [11]. Further witness of the role of EMT as intrinsic to the malignant process is borne by the discovery that beyond specific EMT-inducing factors, such as the core EMT transcription factors Snail and Slug, a multitude of general cancer-regulating pathways are important EMT regulators [9]. Examples specifically pertinent to breast cancer include Her2/Neu receptor-activated pathways as well as ER and PR receptors, discussed in subsequent sections. Due to the incompleteness of the EMT process in cancer, neoplastic cells may more readily revert back to an epithelial state through the reverse process of Mesenchymal to Epithelial Transition (MET), which participates in metastasis establishment in remote organs [12]. The EMT circuitry in transformed cells closely co-operates with the pluripotency network of cancer stem cells in order to obtain the required plasticity for alternating epithelial and mesenchymal states [13].

Several pathways activated in cancer have the ability to activate a set of core EMT transcription regulators which eventually lead to E-cadherin down-regulation and cell-cell adhesion dissolution. EMT core factors include Snail1 and Snail2 (also called Slug), ZEB1 (also known as TCF8 or δEF1) and ZEB2 (also known as SIP1 or Zfhx16), Twist, FoxC2, TCF3 (also known as E47 or E2A), Goosecoid homeobox (also called SAMS) and LBX1.

Although overlapping, the genomic effects of different EMT factors are distinct [14]. In breast cancer, the expression of EMT core factors supports different phases of EMT. For example, involvement of Snail1 appears to be instrumental in the initiation of the process while Twist1 becomes essential later during EMT establishment [15]. ZEB proteins are additional factors required for EMT maintenance [16]. Most information on EMT transcription in breast cancer refers to the triple-negative basal-like sub-type associated with BRCA1 mutations which increase Slug protein stability [17] but data on ER-positive cancers are also available. 

## 3. SNRs and EMT in Breast Cancer

SNRs constitute a sub-family of the nuclear receptor family which has 48 members in humans [4]. The sub-family has six members with differing importance in breast cancer. While ERα is unarguably the most extensively studied protein in the disease and has the distinction of being the first successfully targeted by a treatment protein with the introduction of tamoxifen 40 years ago, PR and AR also have recognized but more controversial roles. ERβ has the peculiarity that, despite being a target of treatment, given that current hormonal therapies inhibit its activity in parallel with the activity of ERα, it is not currently evaluated clinically as a response marker. GR and, even more, MR, although not completely ignored, have hardly obtained the attention of the other NRs and their clinical role as contributors to pathogenesis of the disease or targets to therapy is less well-defined [18]. All six SNRs bind DNA as dimers and the binding sequences (Response Elements, REs) on the promoters are similar [4]. ERα consensus RE (ERE) consists of two tandem examers with the sequence 5’-AGGTCA-3’ in an inverted repeat configuration divided by a spacer of three nucleotides (IR3). ERβ has a similar binding sequence to ERα but the second examer requirements are less strict in certain positions. The four other NRs use also IR3 configuration in their binding but their recognized examer is different from ER in the third and fourth nucleotides and consists of the sequence 5’-AGAACA-3’. In addition, AR and PR may use a direct repeat configuration with a three-nucleotide spacer (DR3) as an alternative to IR3 [19]. Although the presence of a binding site of a SNR is important for the regulation of a target gene, it has to be recognized that a regulation may be indirect through a different transcription factor whose expression is regulated by the SNR. Alternatively, there are occasions where SNRs regulate their targets by being tethered to another transcription factor such as AP-1 or Sp1 [20,21]. Moreover, the presence of a RE is not always sufficient for a regulation to occur, as additional prerequisites have to be fulfilled such as expression of the SNR itself in a given breast cancer cell and a permissive open chromatin configuration obtained at least partially through binding of pioneer factors that guide SNRs to sub-sets of their targets [22].

### 3.1. ERα

**Figure 1:**
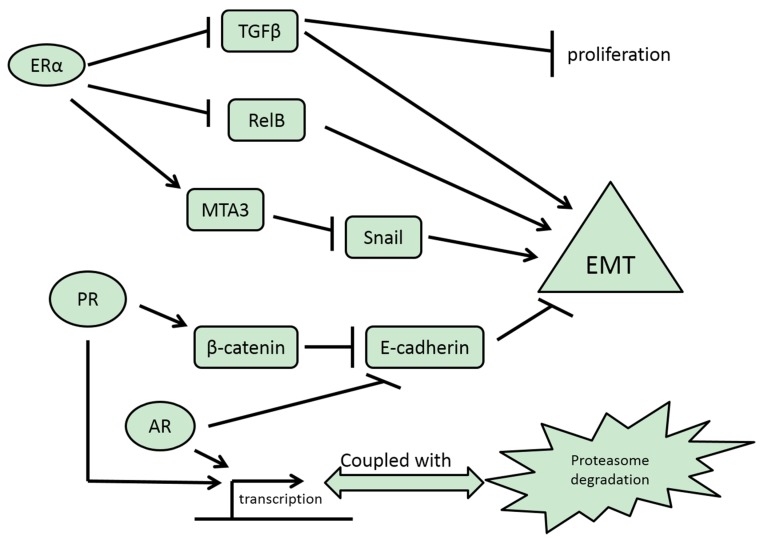
Pathways leading to EMT regulation by SNRs ERα, PR and AR. Transcriptional activity affecting EMT may be coupled with proteasome recycling, and thus the relationship of EMT regulation and receptor expression may not be straightforward. Arrows denote activation and reverse T signs denote inhibition.

ERα is expressed in the majority (about three-fourths) of breast cancers. The degree of expression, though, varies significantly both between different patients with ER-positive disease and in different cells of the same cancer. Currently, all breast cancer patients with ER expression in more than 1% of their cancer cells are treated as ER-positive and hormonal manipulations are included in their adjuvant treatment schedules. An example of differential expression of ER in cells of the same breast cancer is provided by the presence of ER-negative stem cells in ER-positive cancers which mimics the normal epithelial breast tissue hierarchy [23,24,25]. Experiments using human embryonic stem cells expressing ERα and cultured in the presence of estrogens *in vitro* have confirmed that ERα activity is associated with differentiation and promotes the epithelial phenotype [26]. As it will be discussed in next sections, this is due in part to direct suppression of core EMT factors. In addition, ERα suppresses EMT through suppression of EMT, promoting signalling transduction cascades such as TGFβ and NF-κB [27]. Knock-down of ERα by siRNA or by lentiviral-transfected shRNA in ERα-positive breast cancer cells leads to EMT and increased migration and invasion [28,29]. The effect of estrogen-activated ERα on TGFβ signalling down-regulation is mediated by binding to Smad2 and Smad3 and promoting their proteasome degradation [30]. This would impede the growth-inhibiting and EMT-promoting effects of the TGFβ cascade and favour the epithelial phenotype but also accelerate cancer cell growth, both known effects of ERα signalling in breast cancer (Figure 1). Changes in TGFβ signalling between normal and cancerous ER-positive cells have been proposed to explain differences in their proliferation status [31] but may also explain the EMT suppressing effect of ERα in ER-positive cancers. On the other hand, there is an inverse correlation of ERα expression and NF-κB sub-unit RelB expression in breast cancer cell lines and human breast cancer samples [32] (Figure 1). Suppression of ERα in ER-positive MCF7 cells by siRNA leads to up-regulation of RelB and the increased expression is associated with a mesenchymal phenotype, vimentin induction, E-cadherin suppression and increased migration in matrigel assay [32]. Interestingly, the same group has reported a reciprocal regulation whence RelB represses ERα expression [33]. Both Snail1 and ZEB1, which are activated in breast stem-like cells, suppress ER expression [34,35] and in the case of Snail1, NF-κB signalling is involved [35]. Another pathway through which ERα suppresses EMT involves up-regulation of protein MTA3 (Metastatic Tumor Antigen 3) which is a suppressor of Snail and other EMT proteins [36].

### 3.2. ERβ

**Figure 2:**
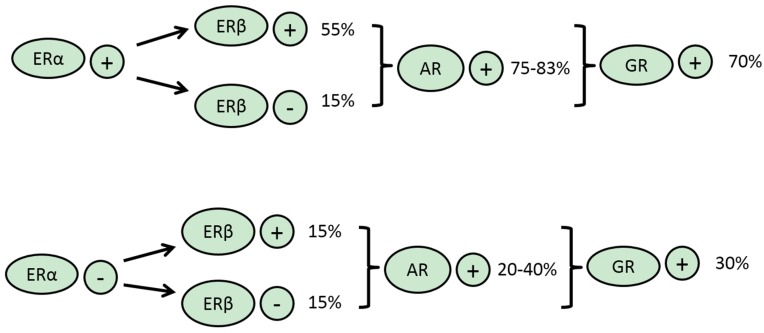
Expressions of other SNRs in ERα-positive and ERα-negative breast cancers.

**Figure 3:**
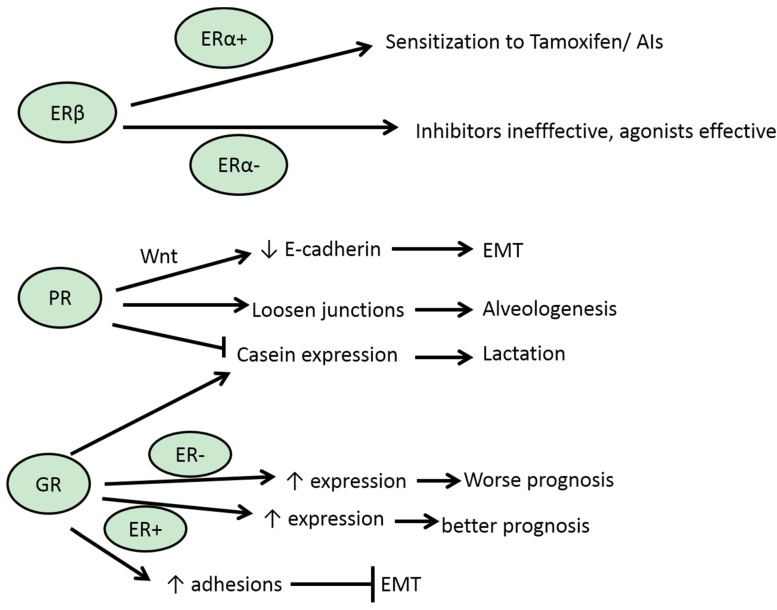
A schematic representation of selected EMT-related effects of ERβ, PR and GR in normal breast development and breast cancer and relationships with ERα status.

ERβ is transcribed from a gene at human chromosome locus 14q23, a different chromosomal location from the ERα gene which is situated at chromosome 6q25 [37]. ERβ is the main receptor expressed in normal mammary epithelium [38]. The two SNRs have very high homology (96%) in their DNA-binding domain and significant homology also in their ligand-binding domain [39]. Thus, they bind natural agonists and drug antagonists with similar affinity. Nevertheless, and despite their high homology, their binding sequence requirements are slightly different, as mentioned. In breast cancer, there is a high correlation of ERα and ERβ expression and most ERα-positive cancers (55% of the total number of breast cancers) also express ERβ (Figure 2). The remaining ERα-positive breast cancers (about 15% of total breast cancers) are ERβ-negative. The ERα-negative breast cancers are equally divided between ERβ-positive and ERβ-negative. In ERα-positive cancers, ERβ has a modulating activity, acting as dominant negative regulator and reducing ERα-dependent transcription [37]. Another immunohistochemical study found only half of luminal cancers to express ERβ, and that was true also for about 40% of basal-like carcinomas [40]. In this study ERβ was a negative prognostic factor for overall survival. Still another study affirmed a somewhat lower ERβ positivity in 33% and 25% of ERα-positive and -negative breast cancers, respectively [41]. In the cohort with ERα-positive cancers that received tamoxifen treatment, ERβ expression was associated with an improved recurrence-free survival. *In vitro* studies confirmed that treatment of ERα-positive breast cancer cell lines with ER inhibitors produced an enhanced inhibitory effect when ERβ was co-expressed [41]. In contrast, ERα-negative cell lines with ERβ expression were not inhibited by ER inhibitors but were inhibited by ERβ-specific agonists (Figure 3). In the same vein, genomic studies have shown that the transcriptomes of the two ER receptors overlap significantly but can be modified by the each other’s presence [42]. Importantly for EMT, in MCF7 breast cancer cells transduced with the receptor, ERβ regulates many components of the TGFβ pathways, resulting in suppression of the TGFβ cascade and up-regulation of the BMP cascade through up-regulation of BMP7 [42]. Overall, these effects result in EMT suppression. A similar genomic study that used another breast cancer cell line, T47D, confirmed only a partial overlap of the two ER receptors transcriptomes but failed to confirm the extensive role of ERβ transcription on TGFβ signalling [43]. Experimental differences notwithstanding, these results may pinpoint the importance of the specific cellular environment for specific gene regulation. The above data of ERβ expression in normal mammary glands as well as the inhibitory effects observed with its activation in the absence of ERα and the suppression of the TGFβ pathway that it mediates argue for a negative effect of ERβ for EMT and a tumor-suppressor and anti-metastatic role in breast cancer. In ERα-positive cancers, the presence of ERβ may, at least in certain occasions, act as an EMT promoter by interfering with ERα activity, but, on the other hand, due to this interference with ERα activity, ERβ may sensitize to hormone-blocking agents by setting ERα activity to a lower level that would be more easy to inhibit.

### 3.3. PR

PR expression is observed mainly in ER-positive breast cancers [44]. There is some controversy about the role of PR in breast cancer, given that progesterone is pro-proliferative in the normal breast and hormone replacement therapy which includes progestagens in addition to estrogens increases the risk of breast cancer in postmenopausal women [45]. Despite this controversy, most data agree that ER-positive/ PR-negative or low breast cancers align with the genomic luminal B sub-type and are associated with inferior clinical outcomes compared with luminal A cancers with high PR expression [46]. Part of the controversy regarding the role of PR in established breast cancer stems also from the fact that PR expression does not completely correlate with its activity and indeed it was proposed that the most transcriptionally active form of PR is unstable due to fast proteasome degradation and would not be captured by standard immunohistochemistry [47] (Figure 1). Besides being a marker of ER transcriptional activity as a target gene of ER, PR may also have profound effects on the ER transcriptome by decreasing the availability of transcription factor AP1, an ER co-factor, by inducing phosphatase MKP-1 (MAPK Phosphatase 1, also known as DUSP1), an inhibitor of the MAPK cascade that activates AP-1 [48]. In addition, this constitutes a negative feedback loop because MAPK positively affects PR transcriptional activity by phosphorylating the receptor at serine 294 residue [49].

A physiologic role of PR in normal mammary glands during pregnancy and lactation in mice is mediated by junctional effects [50]. During pregnancy, high progesterone levels lead to increased PR activity that contributes to suppression of differentiation towards a lactation-capable epithelial specialized cell by suppressing production of milk proteins such as casein (Figure 3). In addition, PR activity helps keep tight junctions between mammary cells loose, a fact that facilitates motility during remodelling of the gland. After parturition, the precipitous drop of circulating progesterone levels leads to both induction of lactation and closure of the tight junctions to allow milk to accumulate in the ducts [51]. Several components of cascades involved in EMT, such as Wnt4 of the Wnt/β-catenin cascade, RANKL, a ligand of the NF-κB cascade and Id4, involved in regulation of EMT factor TCF3/E47, are induced by PR, contributing to the physiologic role of PR during mammary alveologenesis [52,53,54]. These physiologic PR effects may be usurped during EMT of cancer to promote loss of tight junctions and to enable motility and metastasis of transformed cells.

Suppression of E-cadherin and induction of EMT was reported to be mediated by activation of the B isoform of PR (PR-B) *in vivo* in rat mammary tumors and *in vitro* in human cell lines [55]. PR-B is the main isoform in normal mammary development, while PR-A, transcribed from the same gene but from an alternative promoter, lacks the 165 aminoterminal amino acids and acts as a PR-B repressor [56]. Nevertheless, suppression of E-cadherin by PR-B was an indirect effect through activation of the Wnt-β-catenin pathway, a well-known E-cadherin regulator. In contrast, activation of PR with synthetic progestin promesterone had no effect on induction of EMT core factors Snail and ZEB1 [55].

Overall, PR expression and activity favours junctional resolution and EMT. The apparent positive prognostic influence of PR expression in ER-positive cancers may be due to robust ER activity in these cancers, implied by the fact that PR is an ER target gene, and in addition, by the fact that strong PR expression, as detected by immunohistochemistry, may denote, counter-intuitively, a less transcriptionally active protein, given that transcriptional activity is coupled with proteasomal degradation.

### 3.4. AR

AR is expressed in a sub-set of ER-negative breast cancers but also in an even more significant proportion of ER-positive cancers. In a series of triple-negative breast cancers, using a cut-off of 10% to determine AR positivity, it was shown that about 20% of patient tumors were positive for AR [57]. In contrast, another series showed a higher (40%) positivity of AR in triple-negative tumors [58]. A similar percentage was seen in another study which also confirmed that ER-positive cancers had a much higher AR positivity at 83% [59] (Figure 2). A meta-analysis of 19 studies that included 7693 patients showed 74.8% of ER-positive patients to be concomitantly AR-positive while 31.8% of ER-negative patients were AR-positive [60]. In addition, AR expression was a good prognostic marker irrespective of ER expression. 

AR activation by dihydrotestosterone treatment in breast cancer cells has been reported to directly suppress the E-cadherin promoter in an artificial transfection system in breast cancer cell lines and favour metastatic spread *in vivo* in mice [61]. ER-positive cell lines with epithelial morphology displayed a mesenchymal morphology when transfected with AR and treated with dihydrotestosterone. In addition, a binding site for AR was characterized in the E-cadherin gene promoter (Figure 1). Thus, AR may have EMT-promoting effects through this suppression and even independently of any effects on EMT core transcription factors. These results are in contrast with the above-discussed absence of direct induction of E-cadherin by PR, despite the similar binding sequence requirements of the two steroid receptors, and speak again for the importance of the many additional factors regulating transcription initiation that are required in order for transcription to proceed. In addition, even subtle deviations in certain nucleotide sites of binding sequences may affect the binding of one receptor to a specific promoter more than the binding of another [19].

Interestingly, there was no correlation of AR with E-cadherin expression or DFS (Disease-Free Survival) in the aforementioned study of triple-negative cancers [57]. In addition, the fact that AR expression is associated with a good prognosis in all sub-types of breast cancer may imply that it has tumor-suppressing and thus anti-metastatic and EMT-suppressing effects. Nevertheless, as evidenced from the case of ER which has tumor-promoting effects, despite its expression in less aggressive breast cancers and EMT-suppressing activity, this may not be entirely correct. Moreover, AR transcription is associated with turnover of the receptor in the proteasome, similarly to PR activity [62]. Thus, a higher expression by immunohistochemistry may imply higher stability due to lower transcriptional activity. 

### 3.5. GR

GR displays both anti-proliferative and anti-apoptotic effects in mammary cancer and pre-cancerous cells [63,64]. GR expression is seen in about 60% of breast cancers and appears to be associated with ER expression with about 70% of ER-positive tumors expressing GR compared with only about 30% of ER-negative tumors [65] (Figure 2). A gradual decrease of GR expression from normal breast tissue to *in situ* carcinoma to invasive carcinoma has been described [66,67]. GR is able to bind to ER promoter sites in a manner facilitated by FoxA1 and AP1, and displace ER and repress promoter activity [68,69]. These effects are dependent on both ER and GR receptor ligation and may have important implications for glucocorticoid influence on the global ER program and also specifically on EMT in ER-positive cancers. A mutual modulation of ER and GR DNA binding has been confirmed in another genomic study which also confirmed a role for AP1 in these interplays [70]. Regarding ER-negative carcinomas, functional disabling of GR signalling is evident in tumors with BRCA gene mutations or BRCA dysfunction [71]. BRCA1 has a role in the suppression of Twist and its silencing leads to Twist de-repression and EMT [72]. In addition, BRCA1 is a post-translational suppressor of Slug [73]. Thus, EMT promotion in triple-negative basal-like breast cancer cells that have BRCA1 dysfunction may be independent of GR. Nevertheless, EMT was also identified as one of the primary processes regulated by GR in a ChIP-seq study of ER-negative MCF10A breast cancer cells immortalized by c-myc transfection [74]. Interestingly, meta-analysis of data from publicly available, clinically annotated transcriptome studies showed that the prognostic information of GR expression was discordant between ER-positive and -negative patients. In ER-negative patients higher GR expression conferred a worse prognosis while in ER-positive patients the reverse was true [74] (Figure 3). 

During lactation GR co-operates with prolactin-induced transcription factor Stat5 to induce transcription from the casein promoter [75]. In this respect GR is antagonistic to the action of PR which, as mentioned above, suppresses lactation (Figure 3). Whether this is true for other actions of PR, such as its effects on intercellular junctions that affect cell motility, remains to be confirmed. A study with MDA-MB-231 breast cancer cells that are ER- and PR-negative but GR-positive suggests that this may be the case in some cellular environments as treatment with corticosteroids leads to an increase in focal adhesions and a cobblestone-like morphology, implying a MET effect in this setting [76]. This is also in contrast to what was shown in MCF10A cells as discussed above, but argues again for the importance of cellular context. MDA-MB-231 cells line belongs to a sub-set of triple-negative breast cancer cell lines that have been genomically characterised as mesenchymal as opposed to other triple-negative cell lines, such as MDA-MB-468, which are characterised as basal-like [77]. These latter cell lines have been reported to be less sensitive to GR inhibition, possibly due to the functional disabling of the steroid receptor occurring with BRCA1 dysfunction [78]. These studies suggest that GR may have divergent effects depending on ER expression in breast cancer. An additional important result to derive from these data is that promotion of EMT or MET by a transcription factor in a specific setting is not directly related to better or worse prognosis, respectively, but could rather be associated more with facilitation by the factor in question of the passage of a malignant cell from one state to the other in order to be able to metastasize and establish itself in the metastatic site [79].

### 3.6. MR

MR has overlapping effects with PR and GR in breast cancer and can substitute for GR during mammary development [76,80]. Thus, it is expected that it may modulate EMT, although this has not been specifically studied. In kidney epithelial cells, where its main physiologic role resides, stimulation of MR with aldosterone has been implicated in promotion of fibrosis, a hallmark of EMT in tissue injury [81]. Down-regulation of E-cadherin and up-regulation of Smooth Muscle Actin (SMA) were observed after ligand aldosterone exposure in human proximal tubule cells [82], but whether a similar regulation occurs in the breast has not been studied. This would be opposite to the GR effect despite their similar DNA target sequences and reinforces the fact that extrapolations to different cellular contexts are not justified given the high influence of these contexts for the final outcome of a nuclear receptor’s actions. 

From these data it becomes apparent that SNRs have regulatory roles in EMT and affect the process both positively and negatively with implications for the promotion of the reverse MET process as well. The role of ERα in EMT suppression is mediated by both indirect mechanisms but also direct effects on promoters of core EMT factors which will be discussed in subsequent sections. Among the other SNRs, effects are divergent depending on ERα expression. 

## 4. Snail1 and Slug and Regulation by SNRs

Snail1 and Slug (Snail2) are zinc finger-containing transcription factors of the C2H2 type that promote EMT by suppressing expression of E-cadherin and other adhesion molecules. Both proteins bind E-box sequences with the consensus 5′-CANNTG-3′ in the promoter of the E-cadherin gene with their zinc fingers [83]. Snail1 is a more potent E-cadherin suppressor. Both Snail proteins as well as TCF3 are expressed in the branching sites during normal mammary morphogenesis and are necessary and sufficient for induction of the process [84]. Snail1 and Slug protect cells in mammary tubules undergoing branching from apoptosis induced by p53 and BID [84]. Snail1 also protects non-transformed human mammary epithelial cells from anoikis [85]. Snail1 inhibition, on the other hand, correlates with down-regulation of RhoA, a GTPase that promotes motility [86]. Both Snail family members are expressed in breast cancer at the mRNA and protein level [13,87,88]. 

Activation of ERα by estrogen ligation in breast cancer cell lines leads to direct repression of the Slug promoter as the liganded nuclear receptor attracts a repressor complex which includes histone deacetylase 1 and N-CoR (Nuclear Co-Repressor) but not SRC-3 (Steroid Receptor Coactivator 3) or IKKα in MCF7 breast cancer cells [89]. Three ERα half binding sites have been identified in the Slug promoter, but it remains unknown whether one or more of them is the actual required binding site. Of note, a search for full sites in the three human promoters of Slug listed in TRED (Transcriptional Regulatory Element Database, https://cb.utdallas.edu/cgi-bin/TRED/) did not disclose any ERα-binding sequences. Slug suppression in ERα-transfected breast cancer cells treated with estradiol up-regulates E-cadherin expression and decreases their invasiveness in an *in vitro* assay [90]. In contrast, when ERα was knocked down in cells initially expressing it, Slug was up-regulated and cell morphology was altered to a more fibroblast-like phenotype [90]. In addition to direct suppression, ERα suppresses Snail proteins indirectly through transcriptional repressor MTA3 in the case of Snail and through inactivation of GSK3 kinase in the case of Slug [90,91]. In this last study that showed Slug suppression by ERα, Snail was activated, although this effect could not overrule Slug suppression which led to EMT promotion [90]. Moreover, in another study using a different breast cancer cell line T47D, ERα in co-operation with SRC-3 up-regulated Snail and suppressed E-cadherin [92]. Thus, ERα signalling may have divergent effects in Snail protein regulation, depending on the cellular context and co-activators present. It also has to be considered that Snail proteins are regulated by multiple factors and ERα is not their sole regulator. Multiple parallel regulations lead to clinical cases where high levels of Snails expression are observed in breast cancers with high ERα content [93]. This may additionally be due to the fact that expression of ERα is not always associated with functional transcriptional competence.

AR has a role in EMT induction in benign epithelial prostate hyperplasia cells and this effect appears to be mediated through TGF-β signalling and Snail induction [94]. In prostate cancer cell lines expressing AR, treatment with androgens led to significant induction of Slug within 2 h of treatment, suggesting a direct up-regulation. This effect was not observed in AR-negative lines or after knock-down of AR with siRNA [95]. No experimental data are available to ascertain an effect of AR in Snail family regulation in breast cancer cells. In fact, studies have shown significant differences of the AR actions between breast and prostate cells and, thus, data from the prostate cannot be directly extrapolated to breast cancer [96].

An *in silico* investigation of the human Snail and Slug gene promoters listed in the TRED and EPD (Eukaryotic Promoter Database, www.epd.vital-it.ch) promoter databases was performed using the binding sequences listed in the JASPAR database. The database enlists binding logos for ERα, ERβ, AR and NR3C1 (GR). It does not list PR and MR, but their binding sites are similar to AR and GR, respectively, as discussed previously. This search disclosed several binding sites for ERβ and GR in promoters of both Snail family transcription factors (Table 1). As mentioned above, no full sites for ERα were identified in Slug promoters and only a single site was identified in Snail promoters (Table 1). AR has several putative binding sites in Slug promoters but only a few in Snail. Overall, these data would argue for ERβ and GR (and in the case of Slug, for AR) being putative, more important Snail and Slug regulators than ERα. 

**Table 1:** Number of binding sites of Steroid Nuclear Receptors (SNRs) in the different promoters of EMT core transcription factors Snail, Slug and ZEB1 and ZEB2, listed in the TRED and EPD databases. Progesterone Receptor (PR) and Mineralocorticoid Receptor (MR) appear in parentheses in the Androgen Receptor (AR) and Glucocorticoid Receptor (GR) column headings, respectively, as they have the same binding sites. In the last column, the number of promoters with clusters (more than three) of binding sites for each SNR (ERα/ERβ/AR/GR) is presented.

Factor	Database	Promoter	ERα	ERβ	AR (PR)	GR (MR)	Clusters
Snail	TRED	1	0	3	2	4	0/3/0/2
		2	1	7	1	3	
		3	0	3	1	2	
Slug	TRED	1	0	7	4	4	0/2/2/3
		2	0	6	5	4	
		3	0	0	2	7	
ZEB1	TRED	1	1	7	2	6	0/4/1//5
		2	0	4	3	8	
		3	1	7	2	6	
	EPD	1	0	4	1	5	
		2	0	1	0	3	
ZEB2	TRED	1	0	2	2	4	0/1/3/4
		2	0	2	3	3	
		3	0	3	4	7	
	EPD	1	0	1	1	2	
		2	0	1	7	4	

An additional investigation was performed using the Transcriptomine tool, a nuclear receptor target gene database, offered by Nuclear Receptor Signaling Atlas (NURSA) to interrogate publicly available data from microarray experiments (www.nursa.org). An interrogation of the database for target genes with expression increase or decrease by at least two-fold in human mammary tissues or cell lines disclosed regulations of several EMT core transcription factors in experiments using breast cancer cell lines and manipulations of ERα and ERβ, but no experiments with the other SNRs were available. Results from this interrogation relevant to Snail and Slug are discussed in this section and in appropriate subsequent sections for each core factor wherever significant regulations were present. In MDA-MB-321 ERα-negative cells transfected with ERα, activation by estradiol treatment for 24 h led to Snail up-regulation over 11 times greater than compared with estradiol-untreated cells [97]. Given the exposure timing, this may represent an indirect regulation. Additional experiments were performed with the same cell line transfected with a mutant ERα that displays significantly decreased ability to bind EREs but an increased GRE binding. Activation of these mutant transfectants by estradiol exposure for 2 to 24 h led to an up-regulation of Slug consistently at all time points by 2- to 2.7-folds. In contrast, in MCF-7 cells that express endogenous ERα, knock-down of the receptor led to up-regulation of Slug by 30-fold [28]. A smaller increase of Slug was observed when MCF-7 cells transfected with ERβ were treated with estradiol [42]. These data speak for a possible direct up-regulatory effect of GR for Slug (at least in an artificial transfection system) and a down-regulatory effect of ERα in ERα-positive cells, partially antagonized when ERβ is present.

## 5. ZEB1 and ZEB2 and miR200 Family

ZEB1 and ZEB2 are zinc finger transcription regulators that contain a homeodomain flanked by two zinc finger domains. Similar to the two Snail proteins, they promote EMT by binding to DNA through E-boxes of target gene promoters such as E-cadherin [98]. Their function is inhibited by the miR-200 family of miRs which bind multiple sites on the 3’-UTRs of their mRNAs, leading to mRNA destruction, and promote the inverse process MET [99]. The miR-200 family consists of two clusters transcribed from two chromosomal loci. miR-200a, miR-200b and miR-429 genes are located in human chromosome locus 1p36 while miR-200c and miR-141 genes are in chromosome 12p13 [100,101]. In addition to down-regulation of ZEB1 and ZEB2 mRNAs, they also have several other targets throughout the transcriptome and their action leads to a coordinated maintenance of the epithelial phenotype [102]. There exists a double-negative feedback loop, as ZEB1 binds E-boxes in miR-200 promoters and suppresses their transcription [103]. 

ZEB1 is induced by estrogens with kinetics consistent with a direct regulation of transcription by ERα in various organisms and tissues [104,105]. Nevertheless, it was not induced by estrogens in human mammary cancer cell lines MCF7 and T47D [106]. Additionally, ZEB1 was not induced by estrogen treatment in OVCAR3 and Caov-3 ovarian cancer cell lines, but it was promptly induced after one hour in a different ovarian cancer cell line, OV266 [106]. These data support a cell-dependent ZEB1 regulation by estrogens even in the same cancer type. A study that examined miR200c expression in patients with breast cancer found no correlation of the expression of this miR-200 family miRNA with either ER or PR positivity [107]. A similar percentage of ER-positive and ER-negative patients were expressing high or low levels of miR-200c and the same was true of PR-positive and -negative patients [107]. In another study, though, the expression of miR-200 family mRNAs was decreased in clones of MCF7 cells resistant to the ER modulator tamoxifen and to ER down-regulator fulvestrant [108]. Parental cell line cells were sensitive to these agents and expressed a higher level of these miRs. Thus, an association of miR-200 expression with ER expression and function may be present in some cellular contexts, similarly to ZEB expression.

In a study employing breast cancer cell lines expressing one of the two isoforms of PR, either PR-A or PR-B, ZEB1 was found to be up-regulated specifically by PR-B [109]. This study used an artificial system with transfection of either of the two PR isoforms in a breast cancer cell line not expressing endogenous PR. ZEB1 was induced by 3.6 times when cells transfected by PR-B were treated with progesterone, while, as expected, no up-regulation was seen in cells transfected with PR-A, given that this isoform may not interact with DNA by itself.

ZEB1 is induced by AR in prostate cancer cells and in triple-negative breast cancer cells [110,111]. Similar to the induction by estrogens, there were discrepancies in different cellular environments in breast cancer, with a correlation of AR and ZEB1 expressions being evident in ER-negative but not in ER-positive cells [111]. A reciprocal regulation is present with ZEB1 inducing AR. Response elements of each transcription factor are present in the promoter of the other. Two Androgen Response Elements (AREs) in the ZEB1 promoter are both required for ZEB1 induction by androgens in an artificial promoter transfection system in prostate cancer cells [110]. In contrast, androgens do not induce ZEB1 in an AR-positive prostate cancer cell line. The authors of the study suggest that induction of ZEB1 is seen by androgens in studies employing exogenous promoter constructs because these constructs do not carry the 3’-tail used by miR-200 family members, while the endogenous mRNA species fail to show androgen-induced up-regulation because they are promptly degraded by miR-200s [110].

Evaluation of both ZEB1 and ZEB2 promoters from the TRED and the EPD databases reveals that ERβ, AR and GR have clusters of binding sites in ZEB1 or ZEB2 promoters and may be putative regulators. A single binding site of ERα is present in two ZEB1 promoters and no binding sites are present in ZEB2 promoters (Table 1). Transcriptomine data show up-regulation of both ZEB factors in MCF-7 cells with ERα knock-down [28]. This result, in combination with the discussed-above absence of ZEB induction observed in the same cell line [106], is most consistent with a model of ligand-independent suppressive ZEB promoter occupancy by ERα.

## 6. Twist

Twist is a bHLH (basic Helix-Loop-Helix) transcription factor with a role in mammary development [83]. Twist molecules bind E-boxes in target gene promoters as dimers. Twist1 was detected in a small subset (1%) of patients with breast cancer, in circulating tumor cells [112]. A study of the expression of Twist in breast cancer patients showed over-expression in about half of the patients [113]. A similar percentage of the patients in the study showed high expressions of Snail and Slug. An increased Twist1 expression is associated with decreased ER expression, down-regulation of aromatase enzyme and development of letrozole resistance in breast cancer cells [114]. This study did not address whether Twist targets ER for transcriptional down-regulation, but two other studies showed that, in fact, this is the case [115,116]. Twist1 associates with regulatory elements (E-boxes) on the ESR1 promoter and recruits the chromatin-suppressive apparatus. Histone acetylation and DNA methylation are incurred [115]. As a result, cell lines with high Twist expression were noted to be negative for ER by Western blotting, while the reverse was true for cell lines with low Twist expression. Of interest, promoter methylation is indeed a factor contributing to ER silencing in breast cancer patient specimens [117].

Regulation of ER by Twist may contribute to ER negativity of stem cells in ER-positive cancers and also to the presence of mesenchymal features in ER-negative cancers. The reverse regulation of Twist by ER or by other SNRs has not been reported. An *in silico* investigation of promoters of Twist listed in the TRED and EPD databases disclosed the presence of AR and GR sites but not sites of any of the two ERs (Table 2).

## 7. TCF3 and Id Proteins

TCF3, another bHLH transcription factor, is able to induce EMT in breast cancer cells by directly suppressing E-cadherin through binding to E-boxes of the promoter called E-pal and E3 [118]. The HLH factors of the Id family lacking a basic domain are inhibitors of TCF3 and modulate its effects on EMT creation. Despite this inhibition and the fact that TCF3 has been found to bind the E-cadherin promoter alone, Id proteins contribute to EMT maintenance in breast cancer and are expressed, together with TCF3, in human breast cancer samples, with a higher expression in the basal sub-type compared to the luminal sub-type [118]. Similarly, in another study using a different antibody, Id expression was noted in several metaplastic breast carcinomas but not in carcinomas with the “usual” morphology [119]. These data imply that an optimal level of TCF3 activity is required for maintenance of EMT and Id proteins participate in the regulation of this activity. 

Association of TCF3 and Id family factors with the basal sub-type of breast cancers and their lower expression in luminal types may imply a down-regulation by ER and/or PR, but this has not been specifically reported. The investigation of promoters of TCF3 listed in the TRED database shows clusters of ERβ, AR and GR binding sites (Table 2).

**Table 2:** Number of binding sites of SNRs in the different promoters of Epithelial-Mesenchymal Transition (EMT) core transcription factors Twist, TCF3, FOXC2, Goosecoid and LBX1, listed in the TRED and EPD databases. PR and MR appear in parentheses in the AR and GR column headings, respectively, as they have the same binding sites. In the last column, the number of promoters with clusters (more than three binding sites) for each SNR is presented.

Factor	Database	Promoter	ERα	ERβ	AR (PR)	GR (MR)	Clusters
Twist	EPD	1	0	0	2	2	0/0/0/0
TCF3	TRED	1	0	1	1	2	0/1/1/2
		2	1	3	0	6	
		3	0	1	3	8	
FOXC2	TRED	1	0	1	3	1	0/1/1/1
		2	0	3	2	3	
Goosecoid	EPD	1	0	0	1	1	0/0/0/0
LBX1	TRED	1	0	2	4	1	0/1/3/2
		2	0	3	1	0	
		3	0	2	3	3	
		4	0	0	4	3	

## 8. FoxC2

FoxC2, a member of the Forkhead box family of transcription factors, is involved in EMT induction and the metastatic ability of breast cancer cells [120]. Signalling from the TGFβ cascade and the activity of transcription factors Snail, Twist and Goosecoid led to FoxC2 induction concomitantly with induction of EMT. Further experiments, to clarify the role of FoxC2 in EMT, showed that isolated FoxC2 induction in epithelial cells increased expression of mesenchymal markers such as vimentin and N-cadherin but could not completely suppress epithelial proteins such as E-cadherin [121]. These results argue for a role of FoxC2 in mesenchymal phenotype maintenance after induction through other transcription factors. Interestingly, a genomic study has proposed a role of FoxC2 in the reverse MET process [121]. Cellular localization of the protein possibly plays a deciding role in the outcome, with cytoplasmic localization promoting epithelial traits by protecting E-cadherin from internalization while nuclear localization promotes EMT through transcription activity [122]. Insulin signalling, acting through the PI3K kinase, activates FoxC2 [123]. In breast cancer, PI3K is activated down-stream of Her2 and other signalling pathways and this may have implications for FoxC2 induction and EMT promotion. In contrast, when a serine at position 124 of FoxC2 is phosphorylated by Casein Kinase 2 (CK2), the transcription factor is retained in the cytoplasm and the epithelial phenotype is maintained [124]. The FoxC2 gene has been reported to have a higher expression in the claudin-low sub-type of triple-negative breast cancers compared with the basal sub-type and the luminal or Her2-overexpressing breast cancers [125].

No experimental data on FoxC2 regulation by SNRs have been reported. A search for binding sites shows clusters of ERβ, AR and GR binding sites, but an absence of ERα binding sites in both the FoxC2 promoters listed in TRED (Table 2).

## 9. Goosecoid

Goosecoic (GSC) is a gene which encodes for a transcription factor that is highly expressed during embryonic development at the Spemann organizer, an area of the embryo where the establishment of body plan is initiated in gastrulation [126]. Both the Wnt/β-catenin and the TGFβ signalling cascades are important in Goosecoid induction from distinct promoters during development [127]. TGFβ signalling is also important for Goosecoid induction in human adult breast epithelial cells [126]. Goosecoid mRNA is increased in micro-dissected breast cancer specimens compared with adjacent normal epithelium and the protein enhances tumor cell motility. Another cancer type where Goosecoid has been studied and been found to promote metastatic potential is hepatocellular carcinoma [128]. Part of Goosecoid contribution in EMT is mediated by induction of FoxC2 [129]. 

The influence of SNRs on Goosecoid regulation has not been explored in the literature. The single EPD promoter listed has one binding site for AR and one for GR but no clusters of SNR sites (Table 2).

## 10. LBX1

LBX1 (Ladybird homeobox 1) is a transcription factor whose gene is located in human chromosome 10q24. The mouse homolog participates in myogenesis and neurogenesis during development by promoting migration of dermomyotome precursors over long distances and the formation of skeletal muscles of the limbs [130]. Thus, a role of LBX1 has been sought in cancer metastasis. Indeed, in breast cancer, it has been found to be up-regulated compared with surrounding breast epithelium [131]. This is particularly evident in triple-negative cancers. LBX1 directly up-regulates ZEB1, ZEB2, Snail and TGFB2 but not Twist1 and promotes breast cancer cell migration, implying a role as a master regulator of EMT. 

Although no further studies have been published so far regarding the role of LBX1 in breast cancer, its association with ER- and PR-negative cancers may hint at a regulation by steroid receptors. To investigate this possibility, a search for SNR binding sites in its four promoters listed in the TRED database was performed and it disclosed clusters of putative ERβ, AR and GR sites but not any ERα sites (Table 2).

## 11. Pioneer Factors

Pioneer factors are proteins that bind compact chromatin to facilitate the binding of other transcription factors [132]. Several such factors that work to facilitate SNR DNA binding have been identified. These include FOXA1, AP2γ, PBX1 and GATA3. FOXA1 is the best-studied and will be used as an example of how pioneer factors shape the landscape of SNRs in breast cancer and thus may modulate their influence on EMT. FoxA1 has been found to co-bind with ERα in about half of the target genes of ERα in a whole genome ChIP-on-chip study in breast cells [133]. Elevated FOXA1 expression is associated with a better prognosis and sensitivity to hormonal therapy in breast cancer patients [134]. FOXA1 is also a pioneer factor for AR DNA binding in prostate cancer cells and this function may be preserved in breast cancer expressing AR. An *in vitro* study confirmed this role of FoxA1 in ER-negative, AR-positive breast cancer cells [135]. FoxA1 guided AR binding to a sub-set of its binding sites and the silencing of FoxA1 abrogates this binding and negates the apocrine gene signature associated with AR in these cells. Consistently, in a clinical study of non-metastatic triple-negative breast cancers, co-expression of AR with FoxA1 defines a sub-group of patients with a distinct behavior reminiscent of luminal cancers [136]. In prostate cancer, FOXA1 carries reverse prognostic implications with high expression associated with hormone therapy resistance. As already mentioned, FOXA1 also acts as a pioneer factor for GR. As a result, the binding of pioneer factors may be a prerequisite for binding and transcription function of SNRs but may not be involved as a factor in the decision of which specific factor would be favored on the promoter of a specific gene. In contrast to SNRs and other TFs, pioneer factors may bind to remote sites up-stream of transcription initiation sites of target genes. For this reason, investigation of a possible role of these factors in the regulation of EMT core transcription factors should include longer sequences up-stream of transcription initiation sites. As a consequence, identification of pioneer factor binding sites regulating EMT transcription would require interrogation of several kilobases up-stream of initiation sites. Thus, an *in silico* investigation of EMT factors is not feasible through a promoter database interrogation. Of additional interest in a discussion of EMT, it should be noted that FoxA1 is a transcription activator of E-cadherin expression and could have influence on epithelial maintenance independent of SNRs [137].

## 12. Perspective: EMT and Therapeutic Resistance Development and Reversal

The significance of the EMT process in the pathogenesis of cancer, especially its involvement in the motility and metastasis of cancer in general and of breast cancer in particular, continues to be elucidated. Metastatic potential is an in-built capability of neoplastic cells and, in addition, it is intertwined with the network of pluripotency in breast and other cancers [13,138,139,140]. This association provides cancer cells with the plasticity required to switch from the epithelial state to the mesenchymal state during metastasis and then back to the epithelial state in the metastatic site. EMT is also associated with therapy resistance, which is a major hurdle in clinical oncology [141]. The implications of EMT for drug resistance were confirmed in a study of breast cancer in an *in vivo* transgenic mouse model where mice develop breast cancer with a short delay under the influence of a Her2 transgene [142]. Although cancer cells that had undergone EMT were not instrumental for metastatic establishment in this model, after cyclophosphamide treatment these EMT cells were more resistant to apoptosis and displayed an increased abundance in metastatic foci. In addition, cells with EMT features were less proliferative than other breast cancer cells. Decreased cycling is commonly a feature of stem cells which are also resistant to treatments and also commonly co-express EMT features [138]. These studies confirm the clinical importance of EMT in cancer progression, as resistant-to-chemotherapy (or other therapies such as hormonal treatments) clones represent the ultimate source of cancer treatment failures. Moreover, even at diagnosis, human cancer cells have already undergone selection through survival for several generations against adverse conditions and the host immune system. Thus, primary resistance may already be present, leading to first line treatment failure.

This review discusses the regulation of EMT by SNRs. Regulation by ERα is already exploited therapeutically in breast cancer, but several other steroid receptors present opportunities for therapeutic interventions. A more in-depth understanding of the influence of SNRs on EMT pathways and EMT core transcription factors which establish and maintain the transition and participate in feedback loops that reverse the cell to the epithelial state is a prerequisite to further advance therapeutic manipulations of these receptors in breast cancer. It would also offer the opportunity to introduce combinations of targeted interventions to interrupt EMT establishment based on specific molecular expression profiles. Given that approved medications in clinical use exist for all NSRs, receptor expression profiling in individual breast cancers together with knowledge of their regulation of EMT circuits may allow for a more informed targeted approach, leading to successful anti-metastatic treatment, with the additional advantage of not necessitating the cost and effort of new drug development. 
